# The use of combined remote electronic monitoring and blood concentrations of antipsychotics for assessment of therapeutic adherence in patients with schizophrenia: results of a prospective study

**DOI:** 10.1186/s12888-025-06981-3

**Published:** 2025-05-22

**Authors:** Petr Šilhán, Martin Hýža, Samuel Ambroš, Petr Dostálek, Jana Schwarzová, Tomáš Skřont, Denisa Perničková, Pavla Baarová, Martin Augustynek, Ivana Kacířová, Romana Uřinovská

**Affiliations:** 1 Department of Psychiatry, University Hospital Ostrava, 17. listopadu 1790, Ostrava, Poruba, 708 52 Czech Republic; 2 Department of Clinical Neurosciences, Faculty of Medicine, University of Ostrava, Ostrava, Czech Republic; 3 Department of Cybernetics and Biomedical Engineering, Faculty of Electrical Engineering and Computer Science, VSB-Technical University Ostrava, Ostrava, Czech Republic; 4 Department of Clinical Pharmacology, Institute of Laboratory Medicine, University Hospital Ostrava, Ostrava, Czech Republic; 5 Institute of Clinical Pharmacology, Faculty of Medicine, University of Ostrava, Ostrava, Czech Republic

**Keywords:** Schizophrenia, Therapeutic adherence, Therapeutic drug monitoring, Telehealth, Antipsychotics

## Abstract

**Background:**

Schizophrenia is a serious mental illness, the pharmacological treatment of which comprises primarily the use of antipsychotics. However, non-adherence to their use and its reliable determination present a serious clinical and economic problem. This study aimed to determine therapeutic adherence in outpatients with schizophrenia spectrum disorders by combining short-term electronic monitoring of dispenser opening with the measurement of antipsychotic blood concentrations.

**Methods:**

A total of 55 patients underwent a week-long electronic monitoring of dispenser opening and measurement of blood concentrations before and after monitoring. Patients who correctly opened the dispenser at least in 80% of scheduled time points during the weekly interval and, at the same time, did not show a change in blood concentration of the antipsychotic by more than 30% in any direction, were considered adherent.

**Results:**

69.1% of the patients met the adherence criteria, which was less than that determined by the Drug Attitude Inventory (DAI-10), the Visual Analogue Scale (VAS), and the Clinician Rating Scale (CRS). 7.3% of the patients took less than 80% of the prescribed doses and a change in blood concentrations of the antipsychotic by more than 30% was detected in 25.4% of the patients. In 70.9% of patients, the detected concentrations were within the recommended therapeutic reference interval. The groups of adherent and non-adherent patients did not differ statistically significantly in the severity of their illness as determined by the Clinical Global Impression (CGI), the Personal and Social Performance scale (PSP), and the Positive and Negative Syndrome Scale (PANSS).

**Conclusions:**

The combined method of evaluating adherence in schizophrenia patients confirmed the results determined by other methods. The benefits of this approach are described in the paper.

## Background

Schizophrenia is a fairly common chronic mental disease associated with a serious personal and social burden. Antipsychotics are the mainstay of therapeutic intervention, reducing overall symptoms in acute treatment [1], keeping the symptoms almost unchanged once stabilized [2], and preventing long-term risk of relapse and rehospitalization [3]. However, poor adherence to oral antipsychotics is one of the most important risk factors for relapse [4]. Adherence to antipsychotic medication is relatively low in patients with schizophrenia. A review of 39 articles published between 1981 and 2002 found that the non-adherence rate fluctuated between 4% (long-acting antipsychotics non-adherence) and 72% (study on inpatients), with a median value of 40% [5],. Long-acting injectable antipsychotics (LAIs) are one of the key ways to improve adherence traditionally [6]. Misdrahi et al. stated in their more recent paper a similarly wide but generally higher range from 24 to 90% [7]. Moreover, the inconclusiveness of subsequent epidemiological data led some authors to assume that we are unlikely to reach a stronger consensus in the future [8].

The observed lack of concordance can be at least partially attributed to the differences in operational definitions of adherence, the number of different measurements available for adherence assessment, and a variable length of observation [5]. Medication adherence is defined as “the extent to which a person’s behavior– taking medication, following a diet, and/or executing lifestyle changes, corresponds to the agreed recommendations from a health care provider” [9]. Expert consensus guidelines recommend using 80% of taken medication as a criterion for medication adherence [4]. However, subjective assessment methods, often used in clinical practice, usually overestimate medication adherence and a low rate of consensus among different approaches was documented [10]. Therefore, experts recommend using more objective methods like pill counts, pharmacy records, electronic monitoring, or therapeutic drug monitoring of drug serum levels [4].

With regard to electronic monitoring, three dimensions of adherence were described in patients with schizophrenia spectrum disorders: taking adherence (the number of accesses to drug dispensers divided by the number of prescribed doses), regimen adherence (percentage of days with the full number of prescribed doses taken), and timing adherence (the percentage of doses taken within a defined period of time) [11]. Data from electronic monitoring systems are sufficiently correlated with the number of hospitalizations [12], but it is necessary to take into account that monitored patients agree with adherence supervision, meaning they are cooperative and aware of monitoring. Similarly, therapeutic drug monitoring is also affected by the patient’s behavior prior to the blood draw and assessed concentrations are subject to individual variability [12]. Therefore, even such relatively objective measures of adherence have their own shortcomings and limitations.

To tackle the limitations of the individual objective measures described above, this study used a combined approach of electronic adherence monitoring (EAM) and therapeutic drug monitoring (TDM) to determine therapeutic adherence in schizophrenia outpatients. We aimed to (1) utilize a combination of EAM and TDM to determine therapeutic adherence in outpatients with schizophrenia; (2) investigate group differences between adherent and non-adherent patients in terms of the functional level, illness severity and subjective measures of adherence; (3) assess the mutual associations among adherence/functional status/disease severity scores; and (4) evaluate the usefulness and acceptability of electronic pill dispensers for patients with schizophrenia in the Central European environment (Czech Republic).

## Methods

### Electronic pill dispenser selection

Based on the internet search for available dispensers, three different electronic pill dispensers with audio alerts and remote access to adherence monitoring were selected and purchased. During a period of two months, the selected dispensers were tested by the research team to evaluate user and care provider comfort, available functions, and costs. Based on these criteria, the most suitable dispenser was selected to use in the present study: a one-week box dispenser SimpleMed+^®^ (https://www.vaica.com/simplemed/), containing 7 × 4 squares with individual accesses. It notifies optionally the patient/caregiver/provider of dispenser opening and registers data and statistics on the web interface.

### Participants

The sample consisted of patients between the ages of 18 and 65 diagnosed with schizophrenia or schizoaffective disorder who were treated as outpatients at the Department of Psychiatry of the University Hospital Ostrava. All patients were diagnosed by a board-certified physician using the ICD-10 criteria (World Health Organization 1994). Only patients with an at least a 3-month-long remission, who were using at least one oral antipsychotic and signed the informed consent with participation in the study were included. The assessment of remission was based on clinical judgement of the physician. Patients whose medication had been adjusted within the previous 30 days, patients with intellectual disabilities or dementia, and patients actively abusing any psychoactive substance (with the exception of nicotine) were excluded from the study.

### Procedure

During a routine examination, patients meeting the inclusion and exclusion criteria were invited to participate in a study evaluating the usefulness of an electronic dispenser and its effects on blood concentration of the antipsychotic. Those who agreed to participate were scheduled for an initial assessment within 72 h of giving verbal consent to participate. The timing of the assessment was chosen to correspond with the end of the longest interval between taking individual doses of the used antipsychotic, max. 2 h before the typical time of use. If two intervals of the same length between individual doses were identified, the initial assessment was scheduled to match the opening hours of the psychiatric outpatient center (7 am– 3 pm). Outside opening hours, the assessments were carried out at the inpatient unit. If a combination of several oral antipsychotics was used, the one with the highest chlorpromazine equivalent dose that was not used in the form of LAIs was selected for determining the blood concentration of the given medication.

Patients were asked to bring all their oral medication including the nonpsychiatric ones and the timetable for their use to the initial assessment. During the initial assessment, the patient was fully informed about the aims of the study and the procedure and asked to sign the informed consent, and the physician set up the dispenser using the medication timetable to include all their pills for the next 7 days with a maximum of 4 daily doses. The settings included timing of the typical use of individual doses, standard audio alerts (at the time of planned use + three times in ten-minute intervals in the case of a missed dose), and the time interval during which the use is still considered correct (± 2 h from the planned time). After the initial assessment was finished, the dispenser was activated to start from the next medication dose, and its functions were set for the next 7 × 24 h, with the follow-up assessment scheduled so that they arrive within 2 h before taking the last dose in the dispenser. The participants were instructed not to change their smoking habits during the follow-up.

During the initial assessment, the Drug Attitude Inventory (DAI-10) [13] and the Visual Analogue Scale (VAS) were completed by the patient. The physician completed the Clinician Rating Scale (CRS) of adherence [14] and assessed the patient’s condition using The Positive and Negative Syndrome Scale (PANSS) [15], the Personal and Social Performance (PSP) scale [16], and the Clinical Global Impression (CGI) scoring sheet [17]. DAI-10 is a tool used to assess the attitude of the patient towards prescribed medication, with a range varying from − 10 to + 10. A score > 0 indicates a positive attitude. VAS is a simple line segment ranging 0-100%, such estimating the adherence by simply patient´s marking. A minimum of VAS = 80% is required to determine the patient as adherent according to expert recommendations [4]. The CRS is an easy scale consisting of 7 clinician statements about patient adherence. Higher numbers represent greater adherence and CRS = 5 („Passive acceptance“) was used as a cut-off point. The PSP scale is a simple tool to quantify the funcional disabilities in schizophrenia patients. A clinician assesses four domains and reaches the score of up to 100.All participating physicians underwent joint training in the use of the mentioned scales before starting the research. Upon completion of the initial assessment and dispenser setup, a blood sample was collected at the appointed time to determine the blood concentration of the investigated antipsychotic. The follow-up assessment took place after 7 × 24 h from the initial assessment, with a ± 2-hour interval tolerance. During that assessment, the physician did not need to be present as the follow-up included only drawing a blood sample for the determination of the antipsychotic concentration and the completion of a satisfaction questionnaire regarding the dispenser by the patient. The questionnaire included questions on whether the dispenser led to more regular medication use, whether it could be easily operated, whether the patient would be interested in using the dispenser over the long term, and an overall rating of the dispenser using a Czech school grading system (1 = the best possible rating, 5 = the worst possible rating).

### Adherence determination

Patients who met both adherence criteria, i.e., correctly opened the dispenser in at least 80% of time points during the 7-day monitoring [4] and, at the same time, whose blood concentration of the investigated antipsychotic with the maximum concentration did not change by more than ± 30% compared to the first measurement, were considered adherent. If any of these conditions was not met, the patient was considered non-adherent. The blood concentrations of psychotropic drugs were assessed at the Department of Clinical Pharmacology, Institute of Laboratory Medicine, University Hospital Ostrava, Czech Republic, using a fully validated method (ultra-performance liquid chromatography–tandem mass spectrometry). As found in a previous study by our group, the intra-assay coefficient of variation for the concentrations of antipsychotics and their metabolites is 2.1–8.1% and the inter-assay coefficient of variation is 1.7–8.9% [18]. The limit of 30% was determined as the sum of the upper limits of the two above-mentioned intervals of coefficients of variation, i.e., 16.2%, and expanded to 30% in order to eliminate the potential influence of other pharmacokinetic parameters (such as the exact time of medication intake, individual drug metabolism, diet, smoking variability and other).

### Statistical analysis

Data analysis was conducted using IBM SPSS Version 28. First, participants with incomplete data were excluded from further statistical analysis. Raw data was then used to calculate the percentages of adherence from dispenser data and blood concentrations and determine the overall adherence. Descriptive statistics for subjective measures of adherence, illness severity, functional status, and evaluation of the dispenser were computed for the whole sample as well as the adherent and non-adherent groups separately. One-way ANOVA was then conducted to determine whether the non-adherent and adherent patients differed in terms of measures of adherence scales, illness severity, functional status, and evaluation of the dispenser. Assumptions of the ANOVA test were assessed using a visual inspection of variable distributions and Levene‘s Test for Homogeneity of Variances. Chi-square test of independence was performed to examine the relation between LAIs use and adherence. Finally, Spearman correlations were calculated to investigate associations between individual measures. Confidence intervals (CI) were calculated using the Clopper–Pearson approach. Statistical significance was set at P *<* 0.05.

### Ethical approval

The study was approved by the Ethics Committee of the University Hospital Ostrava, reference number 399/2017. The work was carried out in accordance with the Declaration of Helsinki and good clinical research practice as postulated by the European Agency for the Evaluation of Medicinal Products in 2002.

## Results

In total, 87 patients were invited to participate in the study, out of which 61 agreed and 55 attended both assessments and provided blood samples. Out of the six patients who did not complete the study, one failed to attend the follow-up assessment, two arrived outside of the required timeframe, and three did attend the follow-up assessment but did not provide a blood sample. The mean age of the included participants at the beginning of the study was 39.2 ± 10.3 years, with 40 men and 15 women. Overall, 47 of these patients were diagnosed with schizophrenia as their primary diagnosis (i.e., F20 according to ICD-10; WHO 1994), while the rest were diagnosed with schizoaffective disorder (F25 according to ICD-10). The investigated antipsychotics included amisulpride (2x), aripiprazole (8x), clozapine (8x), fluphenazine (1x), haloperidol (1x), olanzapine (29x), paliperidone (2x), quetiapine (3x), and risperidone (1x). Before the electronic monitoring was commenced, the blood concentrations of the dominant oral antipsychotic were within the recommended therapeutic reference range (TRR) [19] in 39 patients (70.9%, CI 57.1–82.4), below the TRR in 11 patients (20.0%, CI 10.4–33.0) and above the TRR in 5 patients (9.1%, CI 3.0–20.0).

**Fig. 1:**
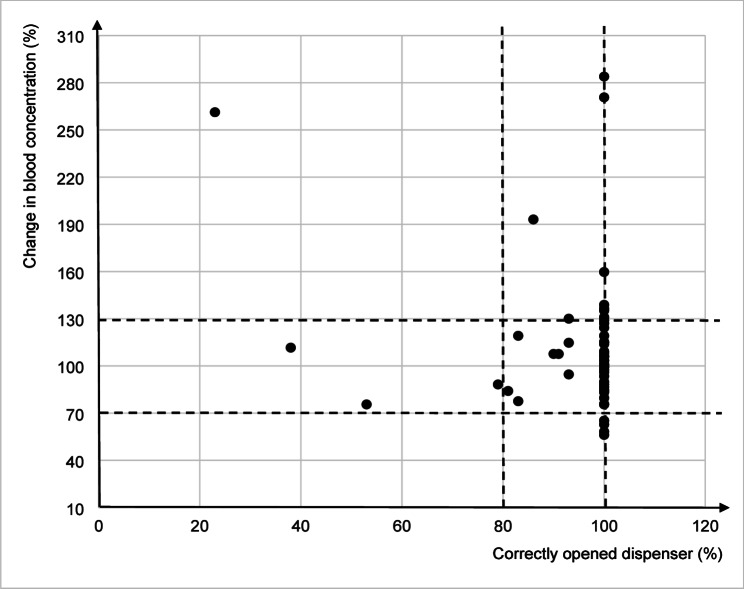
Associations between the percentage of scheduled time points with correctly opened dispenser (EAM) and change of blood concentrations (TDM) of evaluated antipsychotics between examinations. The dashed lines delimit the adherence criteria

During the one-week-long electronic monitoring, the participants opened the dispenser correctly on average in 94.3% ± 15.0% of cases. In total, four patients (7.3%, CI 2.0-17.6) opened the dispenser at the required time on less than 80% of scheduled occasions (38–79%) and were considered non-adherent by EAM. After the one-week-long electronic monitoring, blood concentrations of antipsychotics remained comparable with the initial measurement (original ± 30%) in 41 patients (74.6%, CI 61.0-85.3) and were considered adherent by TDM; in 4 patients (7.3%, CI 2.0-17.6), the concentrations decreased by more than 30% and in 10 patients (18.2%, CI 9.1–30.9), the concentrations increased by more than 30%. A medium association was found between the percentage of dispenser opening and the change in blood concentrations (*r* = 0.490). Electronic adherence and change in blood concentrations of antipsychotics are shown in Table 1; Fig. 1.

**Table 1:** A contingency table showing the associations between the adherence according to dispenser opening at correct times (EAM) and according to blood concentration changes (TDM)

	TDM change < -30%	-30% ≤ TDM change ≤ 30%	30% < TDM change
EAM < 80%	0	3	1
EAM ≥ 80%	4	38	9

After applying the 80% cut-off on the dispenser adherence data and ± 30% change window on the blood concentrations, 38 patients (69.1%, CI 55.2–80.9%) were labeled as adherent and 17 patients (30.9%, CI 19.2–44.8) as non-adherent using the combined method. The average adherence score based on a subjective rating by the clinician (CRS) was 6.0 ± 1.1. With regard to self-reports, the average DAI-10 score was 5.9 ± 4.2 and the average VAS was 90.7 ± 15.9. The average CGI score for the whole sample was 3.8 ± 1.2, PANSS total score 58.9 ± 16.8, and PSP score 60.3 ± 15.6.

**Fig. 2:**
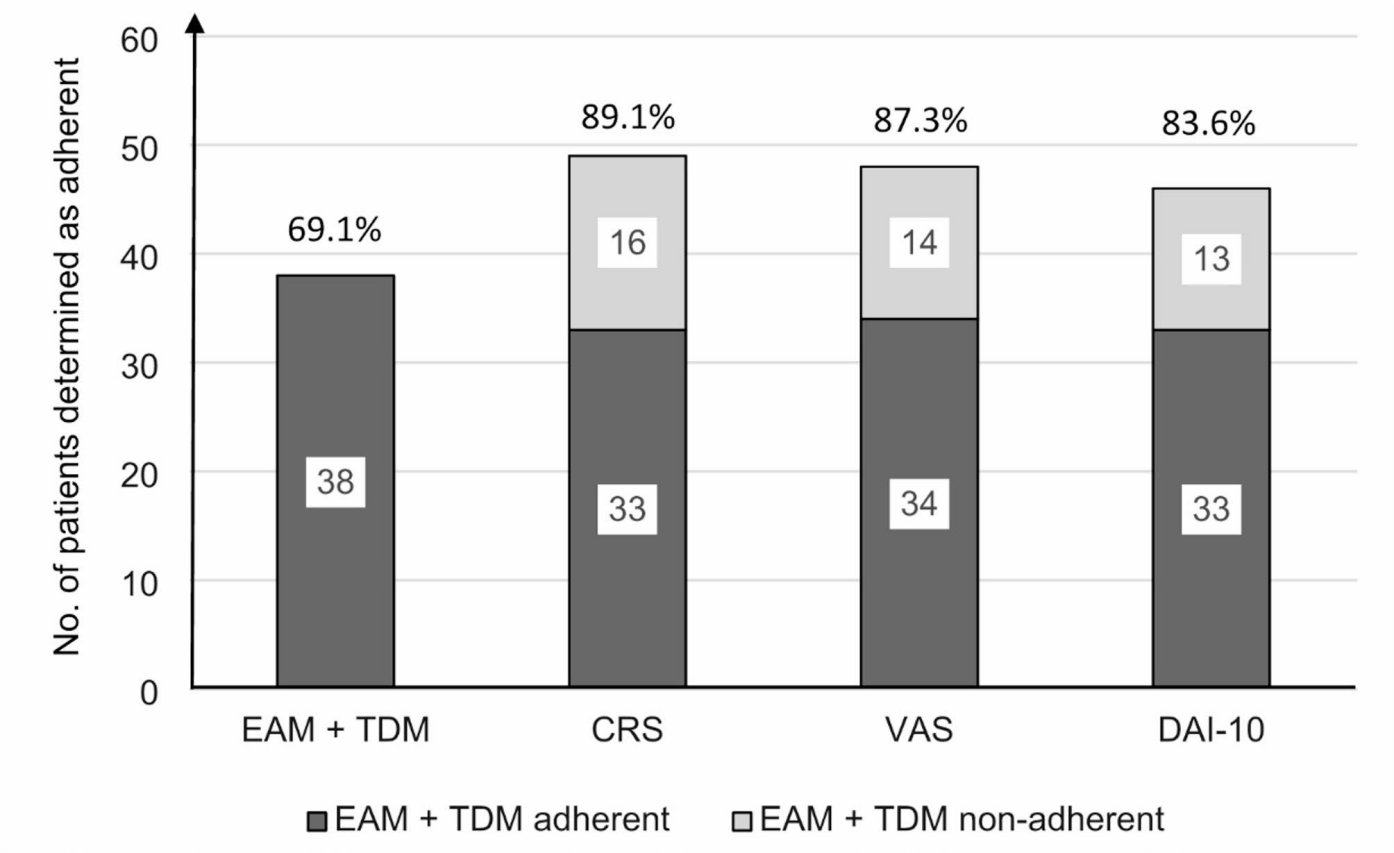
The numbers and percentages of adherent patients determined by individual methods. For each method, it depicts how many of the patients identified as adherent by that method were adherent or non-adherent based on the combined EAM + TDM method

Adherence rates varied depending on the method used to determine them (Fig. 2). The lowest proportion of adherent patients (69.1%) was determined by the combined EAM and TDM method, the highest proportion (89.1%) by the physician´s assessment (CRS ≤ 4). Of the 38 patients considered adherent according to the electronic monitoring and blood concentrations, 5 (13.2%) were determined as non-adherent by the physician (CRS ≤ 4), while by the patients themselves, 4 cases (10.5%) were considered non-adherent according to VAS (VAS < 80%) and 5 (13.2%) according to DAI-10 (DAI-10 ≤ 0). Conversely, among 17 non-adherent patients, the physician assumed adherence (CRS > 4) in as many as 16 cases (94.1%), 14 patients (82.4%) identified themselves as adherent according to the visual-analogue scale (VAS ≥ 80), and 13 (76.5%) patients scored a positive relationship to medication (DAI-10 > 0).

Six one-way analyses of variance (ANOVAs) were conducted to compare whether the adherent group statistically differed from the non-adherent group in individual parameters (Table 2). No statistically significant differences were found between adherent and non-adherent patients in any of the parameters. For this reason, we did not further analyze their predictive value for adherence. However, a trend was observed for the parameters of severity of the condition (CGI) and functional status (PSP), suggesting that adherent and non-adherent patients might differ rather in terms of their functional status than their attitude towards medication.

**Table 2:** Group comparison of adherent and non-adherent patients in terms of their attitude towards medication, clinical impression, functional status, and disease severity using multiple one-way ANOVAs

Measure	Adherent *n* = 38 M (SD)	Non-adherent *n* = 17 M (SD)	F	*p*-value
DAI-10	6.16 (4.20)	5.18 (4.31)	0.633	0.430
VAS	92.18 (9.82)	87.41 (24.70)	1.064	0.307
CRS	6.13 (1.07)	5.76 (1.03)	1.411	0.240
CGI	3.58 (1.80)	4.18 (1.24)	2.935	0.093
PSP	62.76 (15.37)	54.76 (15.13)	3.207	0.079
PANSS	56.84 (16.38)	63.65 (17.11)	1.973	0.166

Note. DAI = Drug Attitude Inventory; VAS = Visual Analogue Scale; CRS = Clinician Rating Scale; CGI = Clinical Global Impression; PSP = Personal and Social Performance scale; PANSS = Positive and Negative Syndrome Scale

Multiple non-parametric Spearman correlations were also conducted to investigate the associations between the individual scales (Table 3). Weak to moderate correlations were identified between the subjective measures of adherence, the strongest correlation was observed between the clinician ratings (CRS) and VAS (*r* = 0.473). Strong correlations were also observed between the measures of function (PSP) and illness severity (CGI, PANSS).

**Table 3:** Spearman correlation coefficients between the measures

	DAI	VAS	CRS	CGI	PSP
DAI					
VAS	0.347*				
CRS	0.331*	0.473*			
CGI	0.065	-0.002	-0.083		
PSP	0.022	0.170	0.273*	-0.608*	
PANSS	0.006	0.061	-0.159	0.659*	-0.739*

Note. DAI = Drug Attitude Inventory; VAS = Visual Analogue Scale; CRS = Clinician Rating Scale; CGI = Clinical Global Impression; PSP = Personal and Social Performance scale; PANSS = Positive and Negative Syndrome Scale; * = *r* ≥ 0.2

In addition to oral antipsychotics, 15 of 55 patients (27.3%) were also treated with LAIs (different from the antipsychotics used to measure blood concentrations). In the LAIs-treated group, 66.7% of patients were adherent to treatment and in the LAIs non-treated group, 70.0%. The difference was not statistically significant (*p* = 0.812).

Overall, 54 patients handed in a complete satisfaction questionnaire regarding the electronic dispenser at the follow-up assessment. Of these patients, 35 (64.8%, CI 50.6–77.2) stated that the dispenser helped them to use their medication more frequently than usual, 52 patients (96.3%, CI 87.3–99.6) considered its operation easy, and 11 patients (20.4%, CI 10.6–33.5) were interested in its long-term use, out of which 4 patients were non-adherent. Using the school grading system, 13 patients (24.1%, CI 13.5–37.4) graded the dispenser as “excellent”, 21 patients (38.9%, CI 25.9–53.1) as “very good”, 12 patients (22.2%, CI 12.0-35.6) as “good”, 7 patients (13.0, CI 5.4–24.9) as “satisfactory” and 1 patient (1.9, CI 0.1–9.9) as “unsatisfactory”; the median grade was “very good”.

## Discussion

The introduction of antipsychotic treatment significantly improved the course and prognosis of schizophrenia. While in the era before antipsychotics and at the time when the disease was recognized by Kraepelin as dementia praecox, three-quarters of patients ended up with severe impairment of function, only a quarter of patients end up with severe impairment of function nowadays (but this impairment is still significantly less severe than in the past) [20]. Use, or rather non-use, of antipsychotics has also short-term serious consequences in the form of relapses, rehospitalization, time to achieve remission and suicide attempts [21].

The patient population participating in our study (*N* = 55, mean age 39.2 years, 72.7% male, mean CGI-S score 3.8, PANSS 58.9 and PSP 60.3) is comparable to other studies on adherence in schizophrenia patients using EAM [7, 22–26].

38 patients (69.1%) met the combined adherence criteria (EAM + TDM); this “double-check” implies that these patients indeed adhered well to their medication regimen. After a week-long EAM, an increase in blood concentrations by more than 30% was observed in nine patients (16.4%) who opened the dispenser regularly during the study; in 1 patient (1.8%), an increase occurred despite insufficient dispenser opening. This indicates a high likelihood of non-adherence of these ten patients before the initiation of the EAM. Three patients (5.5%) showed stable blood concentrations despite insufficient dispenser opening; this suggests that they used the drugs as insufficiently during the study period as before it. Finally, in 4 patients (7.3%), a decrease in blood concentrations by more than 30% was observed despite regular dispenser opening. These patients may have taken the medication excessively before the EAM or in a different daily schedule than they stated, which could have affected the blood sampling times before and after the EAM and, thus, the initial measured blood concentrations.

The observed full adherence in 69.1% (95% CI 55.2–80.9) of patients falls within the wide ranges reported by Lacro et al. (2002) [5], Sendt et al. (2015) [27], or Misdrahi et al. (2018) [7] and is also in accordance with the expert-estimated interval of 51–70% [4]. In one of their early works, Velligan et al. (2007) [22] found full adherence in 63.5% of outpatients based on a 12-week EAM limit of 80% of daily doses. A lower adherence rate in outpatients was found by Remington et al. (2007) [23], where over 4 weeks, only 48% of patients took daily medication in a sufficient dose on 80% of the days; it is, however, necessary to note that the patients in their study were characterized by a higher degree of severity of the condition according to the PANSS. In a study by Yang et al. (2012) [24], 58.8% of patients used more than 80% of daily doses within ± 3 h of scheduled times during the 8-week EAM. Acosta et al. (2013) [25] found during a 3-month EAM that 76% of patients took their daily dose on more than 80% of the days, but when considering also the proper time of use (± 2 h), only 35% of patients took the medication properly on more than 80% of the days. A similar rate of adherence when taking into account only the daily dose was reported by Brain et al. (2014) [26], who found during a year-long EAM that 73% of patients took their daily dose on more than 80% of days. On the other hand, Misdrahi et al. (2018) [7] monitored schizophrenia patients for 6 months after discharge from hospitalization and found that only 37.3% could be considered adherent, while some of the patients did not start taking medication at all after their discharge from the hospital and the proportion of adherent patients continued to decrease over time. In our case, 69.1% of patients were considered adherent based on the combined criterion of opening the dispenser properly within the specified interval (± 2 h) and maintaining relatively stable blood concentrations of the monitored drug. This relatively high rate corresponds rather to the results of studies that did not condition adherence by use within a certain time frame. The difference can be explained by the fact that the dispensers we used repeatedly reminded the patients of the need to take the medication (sound signal), thereby increasing compliance with the regimen. This may also be related to the relatively short study period (7 days), so it was easier to follow the correct regimen during this time.

In 4 patients (7.3%), a decrease in blood concentrations during regular use was observed, which does not exclude the possibility that before the introduction of electronic monitoring, they used higher doses of drugs than prescribed. In a study by Acosta et al. (2013) [25] who performed electronic monitoring of patients with schizophrenia using the MEMS system (the Medication Event Monitoring System), at least one event of overdose with medication was recorded in 74.3% of monitored patients within the 3 months study period; overall, the use of an excessive dose occurred on average in as much as 4.7% of all monitoring days. Yang et al. (2007) [28] evaluated the Medication Possession Rate (MPR), which indicates the proportion of pills picked up from the pharmacy for a given period compared to the number corresponding to the recommended doses. 7.6% of patients with schizophrenia were found to pick up an excessive amount of medication from the pharmacy (MPR > 1.2). These patients did not differ demographically from the adherent group, but they used olanzapine, quetiapine, and risperidone more often than the adherent patients. Valenstein et al. (2002) [29] found that the odds ratio (OR) of hospital admission in patients with MPR > 1.1 compared to the adherent patients (0.8 ≤ MPR < 1.1) was 3.0, which was even more than patients collecting medication less than prescribed (MPR < 0.8) whose OR for hospital admission was 2.4 compared to the adherent group. At the same time, even a small increase in MPR was similarly risky for hospital admission as a relatively significant decrease in MPR (1.1 ≤ MPR < 1.2 corresponded to 0.5 ≤ MPR < 0.6; 1.2 ≤ MPR < 1.3 corresponded to 0.2 ≤ MPR < 0.3). This excessive collection of drugs from the pharmacy could be caused by the deterioration of the condition requiring an increase in medication doses, but also by the disorganization in the use of medication on the side of the patient [29] or less strict monitoring of second-generation antipsychotics by the physicians due to fewer side effects [28]. In any case, the results of our work confirm that a group of patients overusing medication probably exists, and it is necessary to pay attention to it because it can signal the risk of adverse clinical development of the patient’s condition. It should be noted that this medication overuse can be also observed in other groups of patients with mental illnesses, as we have reported in patients treated with antidepressants in the past [30].

No statistically significant difference was found between the group of adherent and non-adherent patients in any scale of adherence or functional status. This is, however, consistent with most of the aforementioned studies [7, 22, 25, 26].

All three scales for the assessment of adherence (CRS, VAS, DAI-10) correlated to a certain degree, i.e. the subjective estimates of adherence by physicians and patients showed a certain degree of similarity. When these scores were used alone to assess adherence, they yielded very similar adherence estimates of around the unrealistic 90%. At the same time, however, these subjective evaluations did not differ significantly between the groups of adherent and non-adherent patients. It is, therefore, possible that the physician accepts to a certain degree the way the patient presents his cooperation and relationship to the medication, regardless of whether it corresponds to reality or not. However, since the mutual correlation was not high, the results rather reflect the inaccuracy of these scales for estimating adherence and the tendency to overestimate it [4]. On the other hand, Brain et al. (2014) [26], who studied patients treated in a community psychiatry setting, reported that adherence according to the subjective assessment by the patient, informant, and staff showed a high degree of agreement with the results of EAM; they explained this by high-quality education of the patients by the staff. Furthermore, the adherence rate determined according to EAM correlated with that determined from the pill count and physician estimate [22], and with a DAI-10 score [24].

LAIs were developed to overcome the high rate of patients´ non-adherence to oral medication, which has been confirmed in real-world studies on different patient populations particularly [31]. Moreover, LAIs treatment reduces the risk of medication discontinuation in cardiometabolic diseases [32]. Regular administration of LAIs leads to more frequent contact of the patient with clinicians which allows for earlier response to patient needs, adverse events, early signs of relapse, and notice medication discontinuation [33]. Thus, it could be expected that patients treated with LAIs would also have higher adherence rates to oral antipsychotics. This assumption was not confirmed in our study because adherence was almost the same in both groups of patients. This may be due to the persistent trend to start LAIs treatment specifically in previously non-adherent patients, but also because of the study conditions, which included primarily cooperative outpatients.

Unlike the most widely used “single-chamber” system MEMS^®^ (https://aardexgroup.com/medication-adherence-packaging/), the “multi-chamber” electronic dispenser used by us (SimpleMed+^®^) supported the dispensing of all medications prescribed to the patient. This is potentially beneficial from the perspective of a stable intake of a complex mix of medications compared to the monitoring of usage of a single drug, as the latter might fail to capture changes in the blood concentrations due to the pharmacokinetic interactions.

A concentration change of 30% was chosen to determine non-adherence. According to our previous experience, this eliminates the variability of the method as well as partial fluctuations in the individual concentration on the part of the patient, including the regimen of the day, times of use and sampling, diet, etc [18, 30]. The same rate of concentration change was also used for assessing non-adherence under controlled use in the study by Velligan et al. 2007 [22].

The omission of more than 20% of the planned doses of medication was the other criterion for determining non-adherence. It was, therefore, possible to completely miss a full day of taking the drug over the 7 days of follow-up with the patient still meeting the criteria for adherence (85.7% of correctly used doses). Such an omission would have likely been detected (based on the blood concentration) if it occurred towards the end of the study period but not if it occurred at its beginning.

Electronic monitoring of dispenser opening remains an indirect way of measuring adherence, as it is not possible to consider it as the use of medication. On the other hand, the determination of blood concentrations is clearly related to the use of drugs, but the individual measurement is complicated by individual metabolic deviations as well as the regimen of medication use in the short interval before the measurement. The combination of EAM and TDM was able to reduce to a large extent the Hawthorne effect, i.e. the change of behavior within the scope of the research project [34]. The research participants were not aware of the detailed research protocol until the moment of consent that was given immediately before the first examination of blood concentrations, and any change in the patients’ behavior during the research influenced by the awareness of EAM was subsequently detectable by a change in blood concentration.

For the sake of completeness, it should be added that in the Czech Republic, by law, all patients are treated free of charge and their treatment is covered by compulsory health insurance, including all antipsychotics in at least one generic form, so access to care is not limited by any financial or other barriers on the part of the patient.

The patients who participated in the study evaluated the usefulness of the electronic dispenser positively, the median was the second-best grade (on a five-grade scale used in the Czech education system). The patients themselves did not find it difficult to use, but on the other hand, only 20% of them reported that they would be interested in long-term use of the dispenser. The causes of this relative reluctance lay in the care of the dispenser (filling it with pills, charging, connecting to the Internet, etc.) as well as in its poorer compactness, which did not allow it to be easily carried away from home.

The small set of patients can be considered a limitation of the study; the number of participants is, however, comparable to previously published studies. It is also necessary to assume that the research methodology including, among other things, the need to train patients to use the dispenser, could have influenced the selection of patients; in effect, patients who were more inclined to cooperate with physicians (and, therefore, more adherent) participated in the research; this “volunteer bias” is, however, an inherent problem associated with this type of studies. Secondly, the very short duration of the study limits its intepretation and relevance for the real clinical practice. Much longer studies are needed both for the verification of the adherence control methods and for the possible long-term improvement of the adherence. Finally, including the determination of metabolites in the adherence assessment would be also beneficial for further improving the accuracy of our approach.

List of limitations.

- small set of patients.
- selection bias (the need to be trained to operate the dispenser).
- short duration of the study.
- absence of determination of plasma levels of metabolites of the relevant antipsychotics.
- including the individuals treated also with long acting injections.

On the other hand, the combined criterion of two parameters that were previously used independently (TDM and EAM), to determine adherence is, to the best of our knowledge, probably the most complex and reliable method employed so far, as it allows assessment of the adherence not only during the study, but also shortly before the study, which partly reduces the “volunteer bias”.

## Conclusions

Therapeutic adherence is an important condition for successful treatment, and, therefore, it is necessary to pay attention to its monitoring in clinical practice. However, the development of reliable and simple methods is a prerequisite for routine use of such monitoring. The approach used in this study, i.e., a combined criterion tested, that is a combination of electronic adherence monitoring and therapeutic drug monitoring, counts among the most complex ones used so far as it allows not only the evaluation of adherence during the study but also prior to the commencement of the study. Employing this objective and direct approach, we have acquired results of adherence (69.1%; confidence interval 55.2-80.9%) similar to those yielded by much more time-consuming methods. As only 55 patients completed the study, the relatively small number of participants is the main limitation. The presented approach, therefore, appears to be promising for studies of adherence monitoring not only in the field of psychiatry but also in other fields of medicine.

## Data Availability

The datasets used and/or analysed during the current study are available from the corresponding author on reasonable request.

## Abbreviations

ANOVA: Analysis of Variance
CGI: Clinical Global Impression
CI: Confidence interval
CRS: Clinician Rating Scale
DAI-10: Drug Attitude Inventory-10
EAM: Electronic adherence monitoring
LAIs: long-acting injectable antipsychotics
MEMS: Medication Event Monitoring System
MPR: Medication Possession Rate
OR: Odds ratio
PANSS: Positive and Negative Syndrome Scale
PSP: Personal and Social Performance Scale
SPSS: Statistical Package for the Social Sciences (statistical analysis software)
TDM: Therapeutic drug monitoring
TRR: Therapeutic reference range
VAS: Visual Analogue Scale
